# Cyr61 Promotes Inflammation of a Gouty Arthritis Model in Rats

**DOI:** 10.1155/2020/8298615

**Published:** 2020-07-24

**Authors:** Mi Zhou, Kan Ze, Liang Hua, Liu Liu, Le Kuai, Ming Zhang, Bin Li, Yifei Wang, Xin Li

**Affiliations:** ^1^Department of Dermatology, Yueyang Hospital of Integrated Traditional Chinese and Western Medicine, Shanghai University of Traditional Chinese Medicine, Shanghai 200437, China; ^2^Institute of Dermatology, Shanghai Academy of Traditional Chinese Medicine, Shanghai 201203, China

## Abstract

**Background:**

Cyr61 is considered a novel proinflammatory factor. Gouty arthritis (GA) is a self-limited inflammatory reaction caused by monosodium urate (MSU) crystals. In this study, we assessed the role of Cyr61 in the inflammatory process of GA.

**Methods:**

We investigated the expression of Cyr61 in MSU-induced rat gout models and MSU-stimulated rat fibroblast-like synovial (FLS) cells. After inhibiting the expression of Cyr61, levels of IL-1*β*, TNF-*α*, and IL-6 were detected by ELISA, qPCR, western blot, and immunohistochemical methods. We probed the downstream NF-*κ*B signaling pathway using the NF-*κ*B inhibitor PDTC, and levels of NF-*κ*B and p-NF-*κ*B were detected by western blot and qPCR.

**Results:**

Our results demonstrate that Cyr61 plays a potent role in the formation of local inflammation in vitro and in vivo. Cyr61 was highly expressed in synovial tissues of gout models, and the expression of Cyr61 protein was also significantly increased in MSU-stimulated FLS cells. Cyr61 promoted MSU-induced acute inflammation via the NF-*κ*B signaling pathway.

**Conclusions:**

Our study has revealed that Cyr61 is an important regulatory factor for the initiation of inflammation in GA. The high expression of Cyr61 protein can induce synovial cells to produce many inflammatory cytokines, such as IL-1*β*, TNF-*α*, and IL-6, partly in an NF-*κ*B-dependent manner. Thus, inhibition of Cyr61 could be a new target and strategy for the prevention and treatment of GA.

## 1. Background

Gouty arthritis (GA) is a recurrent chronic inflammatory disease caused by monosodium urate (MSU) crystals and is the most common inflammatory arthritis among men and postmenopausal women. The prevalence of GA has been rising over the past decades, and epidemiological data have shown a prevalence of 1.4–3.9% [1]. The increase in the prevalence and incidence of GA is mainly due to lifestyle changes, such as high alcohol consumption and purine-rich diets, and is closely related to metabolic syndromes [2].

Acute gout attacks, although characterized by self-limited inflammation, are extremely painful and can lead to joint disability. Hyperuricemia is the greatest single risk factor for developing acute gout. MSU is deposited in the synovium or synovial cavity and appears as microcrystals, which is the basis of acute inflammation. The prolonged deposition of MSU crystals can result in irreversible joint damage with bone erosion and tophus formation [3]. The initial events of MSU crystal-triggered inflammation arise after synovial phagocytes encounter the crystals and phagocytosis occurs [4]. A variety of cells in the human body, including neutrophils, mononuclear macrophages, synovial cells, and T lymphocytes, can react with MSU crystals [5] and release a variety of cytokines.

Although the main cause of gout is well known, many uncertainties persist and its pathogenesis remains poorly understood. For example, the mechanisms behind the preferential deposition of MSU crystals at specific sites and the presence of deposited crystals in joints without clinically apparent inflammation are unclear. The presence of MSU crystals within joints does not necessarily result in an inflammatory attack [6, 7], which suggests that there must be other factors that determine whether the production of inflammatory factors occurs as a result of hyperuricemia.

Recent studies have found that cysteine-rich protein 61 (Cyr61/CCN1) participates in the regulation of the inflammatory microenvironment and is involved in the pathology of inflammation and autoimmune diseases. Thus, Cyr61 is considered a novel proinflammatory factor [8, 9]. Named for its cysteine-rich properties, Cyr61 is an important extracellular matrix protein with proinflammatory functions, which was first isolated from mouse fibroblasts by Lau in 1985 [10]. Under normal circumstances, low expression of Cyr61 can maintain the physiological needs of the body; however, many factors (i.e., inflammatory factors, growth factors, and mechanical tension stimulation) can lead to an increase in Cyr61 synthesis [11–13]. After the highly expressed Cyr61 is secreted into the extracellular matrix, it binds to a variety of integrin receptors on cell membranes and performs a variety of biological functions, such as mediating cell adhesion, migration, proliferation, apoptosis, and angiogenesis [14–16].

Human rheumatoid arthritis (RA) is a chronic, immune-regulated inflammatory disease in which Cyr61 plays an important role. Cyr61 can stimulate synovial cells to secrete high concentrations of IL-6 through the *α*v*β*5/Akt/NF-*κ*B signaling pathway, which promotes the differentiation and proliferation of local Th0 cells into Th17 and the production of IL-17 [17]. Cyr61 can also promote the production of IL-8 in synovial cells of RA patients, which mediates chemotaxis of neutrophils to joints and aggravates inflammation [18]. Furthermore, Cyr61 induces pro-IL-1*β* production in fibroblast-like synovial (FLS) cells through the AKT-dependent NF-*κ*B signaling pathway [19]. However, whether Cyr61 exerts any effect on inflammatory factor production in FLS cells during the immune-inflammatory disease GA has not yet been explored.

In the current study, we used a lentiviral vector (LV) expressing a small hairpin RNA (shRNA) to silence Cyr61 in vitro and in vivo. We found that Cyr61 is involved in the inflammatory process of GA. Cyr61 induced the production of IL-1*β*, TNF-*α*, and IL-6 in rat FLS cells through the NF-*κ*B signaling pathway. These findings indicate that Cyr61 is an important participant in the inflammatory process of GA.

## 2. Materials and Methods

### 2.1. Animals and Experimental Model of Acute Gout (MSU Crystal-Induced Inflammation)

Male Sprague-Dawley (SD) rats (weighing 250 ± 20 g) were purchased from Shanghai Slac Laboratory Animal Co. Ltd. (Certificate No. 2015000552939) and were maintained under specific pathogen-free conditions. All experiments were performed according to the guidelines of the Committee on Protection, Welfare and Ethics of Experimental Animals in Yueyang Hospital of Integrated Traditional Chinese and Western Medicine affiliated to Shanghai University of Traditional Chinese Medicine (no. 18816).

An acute GA model was induced by a technique modified from that of Coderre and Wall [20]. In brief, rats were anesthetized with 1% pentobarbital sodium (40 mg/kg, i.p.), the syringe was inserted into the ankle joint cavity at an angle of 45° to the tibia from the lateral side of the right ankle joint, and 0.2 ml of MSU suspension (2.5 g/100 ml) was injected. In the normal control group, the right ankle joint was injected with the same amount of normal saline. Contralateral bulging of the joint capsule was considered a successful injection.

### 2.2. The Establishment of the Lentiviral Vector Carrying Code for Cyr61 shRNA

The lentiviral vector expressed green fluorescent protein (GFP), and rat Cyr61 shRNA was constructed by Genechem Co. Ltd. (Shanghai, China). An shRNA sequence that targeted the rat Cyr61 sequence (GenBank NM_031327) was designed as follows: 5′-GAGGAATGGGTCTGTGATGAA-3′ (Cyr61). The lentivirus-GFP (LV-GFP) that expressed only GFP was used as a blank control. The randomly selected nonsense sequence 5′-TTCTCCGAACGTGTCACGT-3′ (Cyr61) was used as an additional negative control. The sequences of all of the constructs were confirmed by sequencing. The recombinant lentiviruses encoding Cyr61 shRNA and negative controls (LV-GFP and LV-Cyr61) were prepared to a titer of 7*E* + 8 transfection units (TU)/ml.

### 2.3. Animal Grouping and Administration Method

After adaptive feeding for one week, 20 SD rats were randomly divided into four groups (5 rats per group) as follows: normal control group, gout model group, lentivirus group, and colchicine group. In the lentivirus group, the microinjection of lentiviral vectors was performed as previously described [21, 22]. Rats received injections (2 *μ*l lentivirus solution, titer 1*E* + 7 TU/ml) into the articular cavity after model establishment with MSU administration. For the colchicine group, rats were administrated with colchicine (3 × 10^−4^ g · kg^−1^) by gavage once a day for 3 days. An equal volume of distilled water was administered by gavage in the normal control and model groups. At 72 h after MSU crystal administration, all rats were sacrificed to obtain blood and tissue samples. Centrifugation was used to obtain the blood supernatant for ELISA. Joint tissues were removed and stored for further RNA and protein extraction or fixed in formalin for histopathologic analysis.

### 2.4. Joint Swelling Evaluation

The joint swelling was examined with a Kawasaki Mitutoyo (Mitutoyo, Kawasaki) digital caliper, and the minimum accuracy was 0.01 mm. Joint inflammation was expressed as the ratio of the perimeter of 0.5 mm under the ankle of the right hindfoot to that of the normal control; values exceeding 1.10 were categorized as inflammation.

### 2.5. Histological Studies and Immunohistochemistry

Rat joint tissue sections were prepared and stained with H&E. The expression of Cyr61, IL-1*β*, TNF-*α*, and IL-6 in synovial tissues was determined by immunohistochemical analysis and with the following antibodies: mouse anti-CCN1/Cyr61 (diluted: 1 : 200; Abcam, Cambridge, MA, USA), rabbit anti-IL-1*β*, rabbit anti-TNF-*α*, mouse anti-IL-6 (diluted: 1 : 150; all from BioTNT, Shanghai, China), and the secondary antibodies goat anti-mouse IgG HRP and goat anti-rabbit IgG HRP (diluted: 1 : 2500). Under the same experimental conditions as the negative control, each sample was diluted and incubated with the same type of antibody. Images were acquired using a light microscope (Olympus, Tokyo, Japan) and analyzed with the image processing software (Image-Pro Plus 6.0).

#### 2.5.1. IHC Average Optical Density Value Analysis Method

Each slice in each group was randomly selected with at least 3 200x fields of view for photography. The same brown color was selected as the unified criterion for judging the positivity of all photos by using Image-Pro Plus 6.0. The cumulative optical density value (IOD) and the pixel area (AREA) of each photo were obtained by analyzing each photo. The average optical density value (AO value) is calculated as AO = IOD/AREA; the higher the AO value, the higher the positive expression level.

### 2.6. Synovial Cell Culture and Identification

Synovial tissue specimens were obtained from rat ankle joints. Synovial cells (mainly fibroblast-like synoviocytes) were isolated from tissue explants and cultured as previously described [23]. Briefly, each sample was cut into small pieces and placed into a digestion solution containing collagenase (type I) (Sigma) for 1–2 h with brief mixing. After centrifugation, ethylenediaminetetraacetic acid (EDTA) (Solarbio) was added to the precipitate for digestion for 20−30 min, and the digestion was terminated when single or dispersed cell masses were observed under the microscope. Then, the cells were collected and cultured in Dulbecco's modified Eagle's medium (DMEM) (HyClone) containing 10% fetal bovine serum (FBS) (Sigma) and 1% streptomycin and penicillin (Solarbio) at 37°C with 5% CO_2_. After overnight incubation, nonadherent cells were removed, and adherent cells were seeded into 6-well culture plates (3 × 10^5^ cells/ml/well).

All experiments were performed with cells obtained after passage 3. Cell-specific markers were used to identify synoviocyte populations. FLS cells were vimentin+ and CD68-, as demonstrated by specific fluorescence antibody staining (Abcam). FLS cells were treated with or without MSU (100 *μ*g/ml) separately for 48 h. FLS cells and supernatants were harvested and frozen at -80°C for later cytokine analysis by qPCR and western blot.

### 2.7. Assessment of Cytokines Secreted by Cyr61-Stimulated FLS Cells

The serum concentration of the FLS cell media was gradually reduced (10% FBS → 5% FBS 2 h → 2.5% FBS 2 h) in order to reduce the interference of serum during the experiment. FLS cells were treated with MSU (100 *μ*g/ml) for 24 h; then, exogenous Cyr61 protein (1.20 *μ*g/ml) was added to the samples for 6 h. For controls, Cyr61 protein (20 *μ*g/ml) was added to FLS cells without MSU stimulation for 24 h. The mRNA and protein levels of the cytokines IL-1*β*, TNF-*α*, and IL-6 were detected by qPCR and ELISA.

### 2.8. Immunofluorescence

Cells were washed three times with PBS and treated with 1% Triton X-100. Next, cells were incubated with the anti-p65 antibody (Abcam, 1 : 200) at 4°C overnight and then with an FITC-conjugated goat anti-mouse antibody (Cell Signaling Technology, 1 : 100) at 37°C for 1 h. Cells were counterstained with DAPI (Beyotime Biotechnology, Shanghai, China) to visualize the nuclei.

### 2.9. Probing of the Signaling Pathway Involved in Cyr61-Induced IL-1*β*, TNF-*α*, and IL-6 Production

A special inhibitor of the NF-*κ*B signaling pathway was used to analyze Cyr61-induced IL-1*β*, TNF-*α*, and IL-6 production. Briefly, 4 *μ*M pyrrolidine dithiocarbamate (PDTC, an inhibitor of NF-*κ*B activation; MedChemExpress) was added to the cell culture medium together with 20 *μ*g/ml Cyr61 for 4 h. The expression of IL-1*β*, TNF-*α*, and IL-6 mRNA was then determined using real-time PCR, and the concentrations of IL-1*β*, TNF-*α*, and IL-6 in the culture supernatant were measured by ELISA. The activation of NF-*κ*B was analyzed using western blotting, with specific primary antibodies against NF-*κ*B and phosphorylated NF-*κ*B (Cell Signaling Technology, Danvers, MA, USA).

### 2.10. Real-Time PCR Analysis

Total RNA was extracted from tissues or cells using the TRIzol reagent (Invitrogen, Carlsbad, CA, USA), and messenger RNA (mRNA) was converted to cDNA using a cDNA reverse transcription kit (Fermentas). Real-time PCR was performed using SYBR Green Master Mix (Thermo, Eugene, OR, USA) according to the manufacturer's instructions. The primers used in this study were as follows: Cyr61: forward 5′ AAAGGTCTCCTGGGTTTC 3′ and reverse 5′ ACTGCGTTACTGTCCATC 3′; IL-1*β*: forward 5′-TGTGATGTTCCCATTAGAC-3′ and reverse 5′-TCTTTGGGTATTGTTTGG-3′; TNF-*α*: forward 5′-CCACGCTCTTCTGTCTACTG-3′ and reverse 5′-GCTACGGGCTTGTCACTC-3′; IL-6: forward 5′-GTTGCCTTCTTGGGACTG-3′ and reverse 5′-ACTGGTCTGTTGTGGGTG-3′; NF-*κ*B1: forward 5′-CTGCTTACGGTGGGATTG-3′ and reverse 5′-TTGCTTCGGTCTTGGTGC-3′; and GAPDH: forward 5′-GGAGTCTACTGGCGTCTTCAC-3′ and reverse 5′-ATGAGCCCTTCCACGATGC-3′. All primers were validated according to the protocol. Data were collected, and quantitative analysis was performed using an ABI Prism 7300 sequence detection system (Applied Biosystems, Foster City, CA, USA). GAPDH was chosen as the internal loading control for standardization between samples, and relative mRNA levels of target genes were calculated by the 2^-*ΔΔ*Ct^ method.

### 2.11. Western Blot Analysis

Equal amounts of whole joint tissue were lysed in lysis buffer, and tissue proteins were extracted. The cells were lysed, and the supernatant was used for protein quantification. Protein levels in different groups were expressed as a ratio to that of the corresponding *β*-actin or GAPDH. We used the following primary antibodies: rabbit anti-IL-1*β*, rabbit anti-TNF-*α*, mouse anti-IL-6 (all from Abcam, Cambridge, MA, USA), rabbit polyclonal anti-Cyr61 (Invitrogen, Carlsbad, CA, USA), rabbit anti-phospho-NF-*κ*B p65, rabbit anti-NF-*κ*B p65, rabbit anti-GAPDH (all from Cell Signaling Technologies, Beverly, MA, USA), and rabbit anti-*β*-actin (BioTNT, Shanghai, China). The secondary antibodies used were goat anti-rabbit IgG HRP, goat anti-mouse IgG HRP, and donkey anti-goat IgG HRP (all from Beyotime Biotechnology, Shanghai, China). The target proteins were examined using a Tanon 5200 imaging system (Tanon Science & Technology Corporation, Shanghai, China) and visualized with an autoradiography film.

### 2.12. ELISA

The levels of IL-1*β*, TNF-*α*, and IL-6 in serum or supernatants were determined using a sandwich ELISA (sensitivity 0.8–300 pg/ml; R&D Systems, Minneapolis, MN, USA) according to the manufacturer's instructions.

### 2.13. Statistical Analysis

All results are presented as means ± SEM. Differences among groups were determined by ANOVA, and comparison between two groups was analyzed by the *t*-test using GraphPad Prism 7.0 software (GraphPad Software, Inc., San Diego, CA, USA). A *P* value < 0.05 was considered statistically significant.

## 3. Results

### 3.1. Cyr61 Is Overexpressed in MSU-Induced Rat Gout Models and FLS Cells

**Figure 1:**
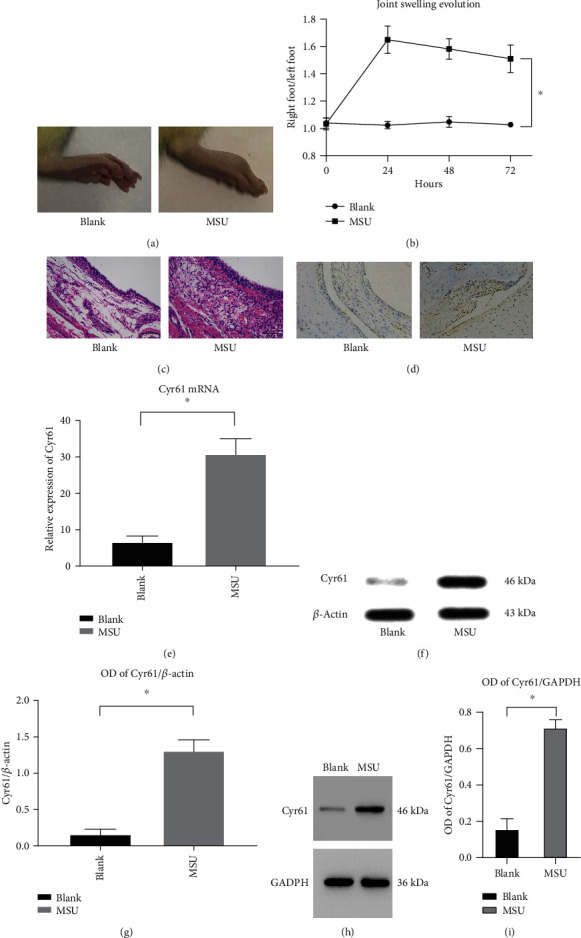
(a) The pictures of arthritis from the blank group and MSU-induced murine gout model group after 72 h. (b) The ratio of the right foot to the left foot (normal control) from 0 h to 72 h (^∗^*P* < 0.05). (c) H&E staining of synovial tissue from the blank group and MSU-induced murine gout model group. (d, f) Immunohistochemical staining of Cyr61, relative expression of Cyr61 mRNA, and the protein level of Cyr61 in the synovial tissue from the same groups (^∗^*P* < 0.05). (g) Protein level in different groups was expressed as a ratio to *β*-actin (^∗^*P* < 0.05). (h) The protein level of Cyr61 in the rat FLS from the same groups. (i) Protein level in different groups was expressed as a ratio to GAPDH (^∗^*P* < 0.05).

Numerous studies have demonstrated that Cyr61 participates in the pathogenesis of inflammatory diseases [24]. To explore the role of Cyr61 in GA, we first constructed a gout rat model using MSU crystals (Figures 1(a)−1(c)). Then, we examined Cyr61 concentrations in synovial tissues from MSU-induced rat models. The results showed that both the protein and mRNA Cyr61 levels were increased (*P* < 0.05) in MSU-induced rat models (Figures 1(d)−1(g)). Simultaneously, we investigated whether Cyr61 could be directly induced by MSU crystals as a danger signal in FLS cells. According to the literature [25, 26] and the results of our previous experiments, we stimulated FLS cells with MSU suspension (100 *μ*g/ml), and the levels of IL-1*β*, TNF-*α*, and IL-6 increased significantly at 48 h. In this experiment, FLS cells were incubated with MSU suspension (100 *μ*g/ml) for 48 h, and both protein and mRNA Cyr61 expression increased (*P* < 0.05) due to MSU stimulation (Figures 1(h)−1(i)).

### 3.2. Blocking Cyr61 Expression Attenuates MSU-Induced Arthritis in Rats

Previous studies have shown that the production and release of IL-1*β* are the first and most important events in gouty inflammation [27]. TNF-*α* can enhance the activity of neutrophils and lead to the mass production of IL-1 [28]. The proinflammatory cytokines IL-6 and IL-1 are the key to initiating innate immune responses [29].

**Figure 2:**
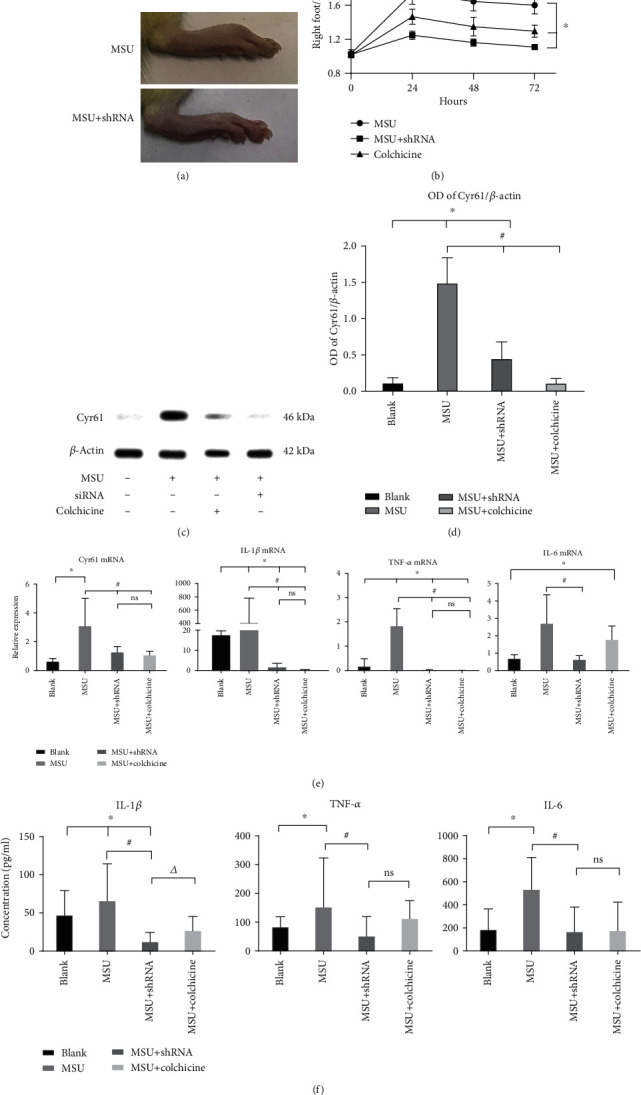
(a) The pictures of arthritis from the MSU-induced murine gout model group and the shRNA group after 72 h. (b) The ratio of the right foot to the left foot (normal control) from 0 h to 72 h (^∗^*P* < 0.05, vs. blank). (c) The protein level of Cyr61 was verified by western blot in the rat synovial tissue from the blank or MSU-induced gouty model groups with or without shRNA interference and colchicine as the positive control. (d) Protein level in different groups was expressed as a ratio to *β*-actin (^∗^*P* < 0.05, vs. blank; ^#^*P* < 0.05, vs. MSU). (e) Relative expression of Cyr61, IL-1*β*, IL-1, and TNF-*α* mRNA in the same groups (^∗^*P* < 0.05, vs. blank; ^#^*P* < 0.05, vs. MSU). (f) The concentrations of Cyr61, IL-1*β*, IL-1, and TNF-*α* were verified by ELISA in the same groups (^∗^*P* < 0.05, vs. blank; ^#^*P* < 0.05, vs. MSU; ^△^*P* < 0.05, vs. MSU+shRNA).

**Figure 3:**
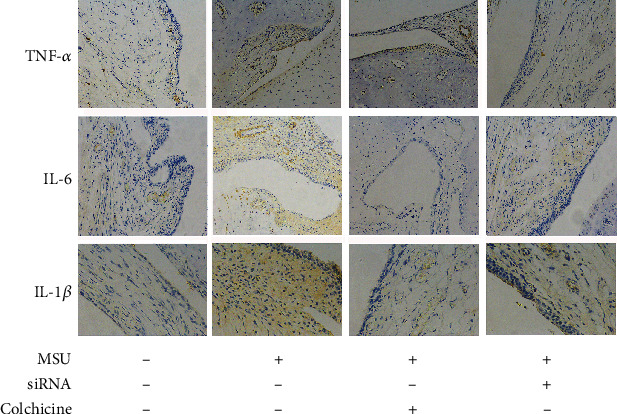
The pictures of arthritis from MSU-induced gouty model groups with or without shRNA interference.

We then used a specific Cyr61 interference lentivirus to knockdown Cyr61 expression in synovial tissues. After MSU crystal administration, amelioration of ankle swelling was observed at 24 h after lentivirus injection. These results suggest that interfering with the expression of Cyr61 has a certain therapeutic effect on MSU-induced arthritis (Figures 2(a)−2(d)). Rats were killed 72 h after MSU crystal injection, and the right ankles were removed to assess joint histopathology. The results showed that IL-1*β*, TNF-*α*, and IL-6 were markedly reduced (*P* < 0.05) in Cyr61-knockdown tissues (Figures 2(e)−2(f)), which is consistent with the immunohistochemical findings of joint tissues. The diseased synovial lining of the model group contained large amounts of IL-1*β*, TNF-*α*, and IL-6, whereas tissues from the lentivirus group contained only small amounts of these inflammatory factors (Figure 3).

### 3.3. Cyr61 Induces IL-1*β*, TNF-*α*, and IL-6 Production in MSU-Stimulated FLS Cells, While Cyr61 Inhibition Decreases These Cytokines

**Figure 4:**
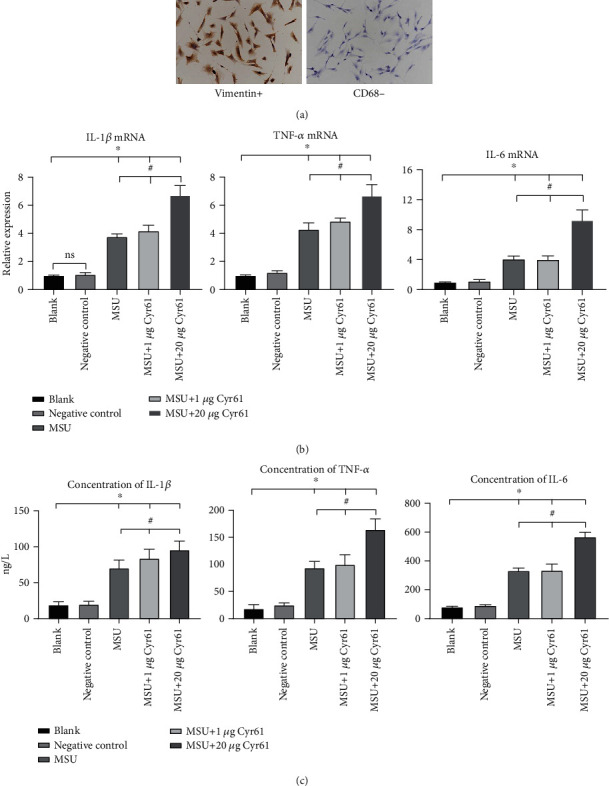
(a) We used vimentin and CD68 as the rat FLS markers. (b) Relative expression of Cyr61, IL-1*β*, TNF-*α*, and IL-6 mRNA in the blank, negative control, MSU-induced, MSU+1 *μ*g Cyr61, and MSU+20 *μ*g Cyr61 groups by PCR. (c) Protein levels of Cyr61, IL-1*β*, TNF-*α*, and IL-6 in the same groups by ELISA (^∗^*P* < 0.05, vs. blank; ^#^*P* < 0.05, vs. MSU+20 *μ*g Cyr61).

Immunofluorescence staining revealed positive vimentin expression and negative CD68 expression in synovial cells, which indicated that the cultured cells were FLS cells (Figure 4(a)). We analyzed the potential effect of Cyr61 on the expression of IL-1*β*, TNF-*α*, and IL-6 in MSU-stimulated FLS cells. In the light of our preliminary experimental results, we stimulated FLS cells with different concentrations of exogenous Cyr61 (1.20 *μ*g/ml) and measured IL-1*β*, TNF-*α*, and IL-6 concentrations by ELISA and real-time PCR. We found that 20 *μ*g/ml Cyr61 significantly stimulated IL-1*β*, TNF-*α*, and IL-6 expression in FLS cells (*P* < 0.05) (Figures 4(b) and 4(c)).

**Figure 5:**
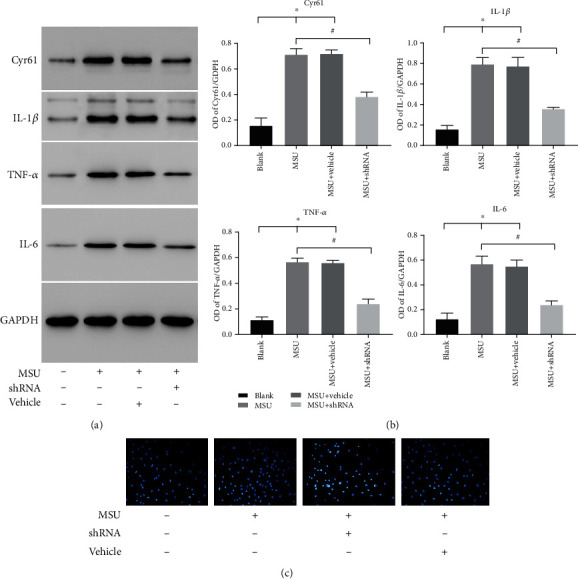
(a) Protein levels of Cyr61, IL-1*β*, IL-6, and TNF-*α* in the blank or MSU-induced groups with or without shRNA interference. (b) Protein levels in different groups were expressed as a ratio to GAPDH (^∗^*P* < 0.05, vs. blank; ^#^*P* < 0.05, vs. MSU-induced). (c) Hoechst staining of rat FLS in the same groups.

To identify the role of Cyr61 in FLS cell cytokine expression, we used a specific Cyr61 interference lentivirus to knockdown Cyr61 expression in FLS cells. FLS cells were treated with both MSU suspension and Cyr61 interference lentivirus. After 48 h, we discovered that both protein and mRNA expression of IL-1*β*, TNF-*α*, and IL-6 were markedly reduced in Cyr61-knockdown FLS cells (*P* < 0.05) (Figures 5(a) and 5(b)). After lentivirus treatment, Hoechst-33258 fluorescent staining revealed that FLS cell nuclei exhibited diffuse homogeneous blue fluorescence, indicating that the lentivirus did not affect the growth of FLS cells (Figure 5(c)).

### 3.4. Cyr61 Promotes MSU-Induced FLS Cell Inflammation by Activating the NF-*κ*B Pathway

**Figure 6:**
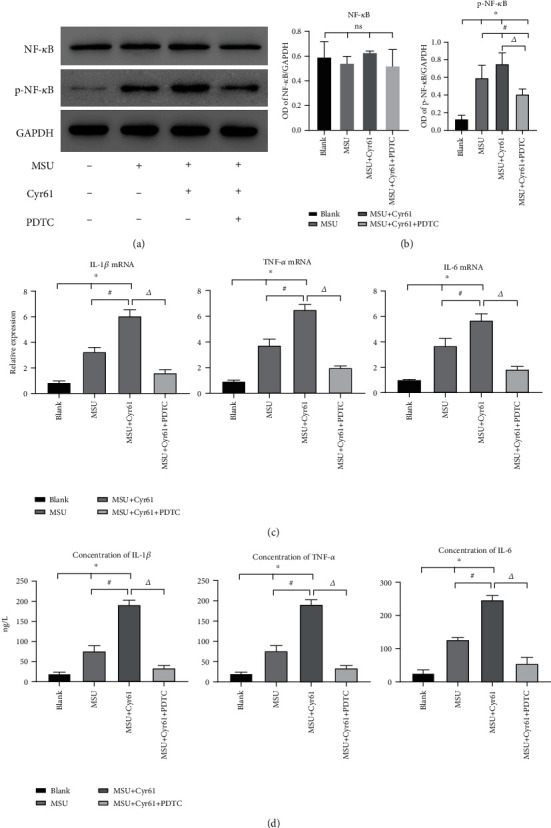
(a) Protein levels of NF-*κ*B and p-NF-*κ*B of rat FLS in the blank or MSU-induced groups with or without PDTC suppression. (b) Protein level in different groups was expressed as a ratio to GAPDH. (c) Relative expression of IL-1*β*, TNF-*α*, and IL-6 mRNA in the blank or MSU-induced groups with or without PDTC suppression. (d) Protein levels of IL-1*β*, TNF-*α*, and IL-6 in the same groups by ELISA (^∗^*P* < 0.05, vs. blank; ^#^*P* < 0.05, vs. MSU-induced; ^△^*P* < 0.05, vs. MSU+Cyr61).

**Figure 7:**
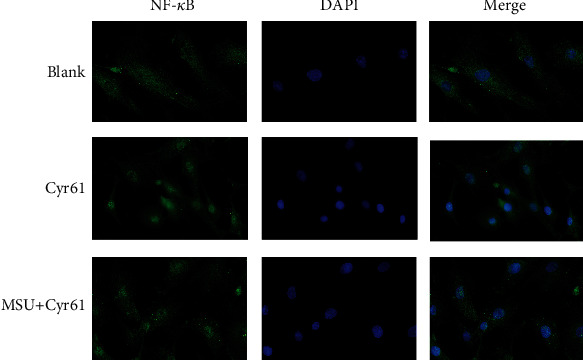
The immunofluorescence of rat FLS in the blank, Cyr61, and MSU with Cyr61 groups.

To investigate how Cyr61 promotes MSU-induced inflammation of FLS cells, we examined proteins related to the NF-*κ*B signaling pathway. MSU alone or costimulation of MSU and Cyr61 did not affect NF-*κ*B, but rather the phosphorylation level of the NF-*κ*B protein (Figures 6(a) and 6(b)). Costimulation of MSU and Cyr61 increased (*P* < 0.05) the phosphorylation level of NF-*κ*B protein, while the addition of PDTC (an inhibitor of the NF-*κ*B pathway) decreased (*P* < 0.05) the phosphorylation level of NF-*κ*B (Figures 6(a) and 6(b)). The expression of IL-1*β*, TNF-*α*, and IL-6 in FLS stimulated with Cyr61 combined with MSU decreased significantly in the presence of the NF-*κ*B inhibitor (Figures 6(c) and 6(d)). An immunofluorescence assay revealed that the level of NF-*κ*B protein nuclear translocation in the MSU-combined Cyr61 group was higher (*P* < 0.05) than that in the group of Cyr61 alone (Figure 7).

These results indicate that Cyr61 promotes MSU-induced inflammation of FLS cells by activating the NF-*κ*B pathway.

## 4. Discussion

GA is an immune-inflammatory disease mediated by the deposition of MSU crystals in synovial tissues, which is a key pathological factor of gout attack. The occurrence of GA inflammation is closely related to IL-1*β*, TNF-*α*, and IL-6. At present, the TLR-MyD88-dependent signaling pathway and NLRP3 inflammasomes are mostly studied in the pathogenesis of GA [30, 31]. However, other regulatory pathways that mediate the production of these proinflammatory cytokines may exist. Discovering such pathways is crucial to comprehensively elucidate the mechanisms of inflammation and tissue injury.

Cyr61 is now considered a novel proinflammatory factor. Whether Cyr61 is involved in the production of inflammatory factors during GA inflammation remains unknown. In this study, we found that IL-1*β*, TNF-*α*, and IL-6 were significantly increased in synovial tissues and serum of MSU-induced rat gout models, which is consistent with the characteristics of the GA inflammatory reaction. More importantly, we also found that Cyr61 was highly expressed in synovial tissues of MSU-induced rat gout models. In order to clarify that Cyr61 is involved in the production of these inflammatory factors, we then used a specific interference lentivirus to knockdown Cyr61 expression in synovial tissues and found that the expression of IL-1*β*, TNF-*α*, and IL-6 was significantly decreased. Colchicine is a classic drug for the treatment of acute gout. Our results showed that colchicine significantly decreased the expression of Cyr61, IL-1*β*, and TNF-*α* protein and mRNA as well as the expression of IL-6 protein. Therefore, we infer that Cyr61 is involved in the inflammatory process of GA, can promote the secretion of IL-1*β*, TNF-*α*, and IL-6, and is an important participant of the GA inflammatory process. To further confirm this idea, we carried out additional in vitro experiments.

Synovial cells are mainly divided into macrophage-like synovial (MLS) and fibroblast-like synovial (FLS) cells, of which FLS cells are characteristic synovial cells [32]. We used MSU suspensions to stimulate rat FLS cells, and the results showed that MSU could induce an increase in IL-1*β*, TNF-*α*, and IL-6 secreted by FLS cells as well as an increase in Cyr61 expression. We then used a Cyr61 interference lentivirus to knockdown Cyr61 expression in FLS cells and found that the production of IL-1*β*, TNF-*α*, and IL-6 also decreased significantly. On this basis, we used exogenous Cyr61 protein to stimulate FLS cells. Cyr61 protein could promote the secretion of IL-1*β*, TNF-*α*, and IL-6 in MSU-induced FLS cells. These results confirm that the Cyr61 protein can aggravate the inflammatory response by upregulating the secretion of IL-1*β*, TNF-*α*, and IL-6 in MSU-stimulated FLS cells. In this process, Cyr61 not only is an extracellular matrix protein but also plays an important role in promoting inflammation.

In order to determine the mechanism with which Cyr61 promotes FLS cells to secrete these inflammatory factors, we evaluated the profiles of NF-*κ*B, a well-known inflammatory pathway. As expected, the NF-*κ*B pathway contributed to Cyr61-induced IL-1*β*, TNF-*α*, and IL-6 production in FLS cells. As a nuclear transcription factor widely present in eukaryotic cells, activated NF-*κ*B can bind to specific sequences in a variety of inflammatory factor gene promoters and can participate in the transcription of inflammatory factors [33, 34]. In this study, costimulation of MSU and Cyr61 increased the phosphorylation level of NF-*κ*B protein and the expression of well-characterized downstream cytokines of NF-*κ*B signaling, whereas inhibiting NF-*κ*B exerted the opposite effects. This suggests that Cyr61 might contribute to NF-*κ*B activation in MSU crystal-induced inflammation.

In this study, through the preliminary analysis of the expression pattern of Cyr61 in the synovium and FLS cells of the GA model rats and the interference of Cyr61 expression by a shRNA lentivirus, the role of Cyr61 in the inflammatory process of GA was explored. It was found that Cyr61 protein, as an important proinflammatory factor, promoted MSU-induced acute inflammation via the NF-*κ*B signaling pathway.

## 5. Conclusion

Cyr61 is an extracellular matrix protein that is significantly related to acute and chronic inflammation and tissue injury. Our study demonstrates for the first time in an experimental GA model in vivo and in vitro that Cyr61 plays an important immunomodulatory role by upregulating proinflammatory cytokines. Cyr61 might represent a novel potential target in GA treatment.

## Data Availability

No data were used to support this study.

## Abbreviations

Cyr61: Cysteine-rich protein 61
DAPI: 4,6-Diamino-2-phenyl indole
DMEM: Dulbecco's modified Eagle's medium
EDTA: Ethylenediaminetetraacetic acid
ELISA: Enzyme-linked immunosorbent assay
FBS: Fetal bovine serum
FITC: Fluorescein isothiocyanate
FLS: Fibroblast-like synovial
GA: Gouty arthritis
GAPDH: Glyceraldehyde-3-phosphate dehydrogenase
GFP: Green fluorescent protein
H&E: Hematoxylin-eosin staining
HRP: Horseradish peroxidase
IKK: I kappa B kinase
IL: Interleukin
LV: Lentiviral vector
MLS: Macrophage-like synovial
MSU: Monosodium urate
NLRP3: Nod-like receptor protein 3
TLRs: Toll-like receptors
TNF-*α*: Tumor necrosis factor-*α*
MyD88: Myeloid differentiation factor-88
NF-*κ*B: Nuclear factor-kappa B
RA: Rheumatoid arthritis
shRNA: Small hairpin RNA
SD: Sprague-Dawley
TH: Helper T cell
PCR: Polymerase chain reaction
PDTC: Pyrrolidine dithiocarbamate.
